# First case report of complete paternal isodisomy of chromosome 10 harbouring a novel variant in COL17A1 that causes junctional epidermolysis bullosa intermediate

**DOI:** 10.1186/s12920-022-01285-x

**Published:** 2022-06-18

**Authors:** Yao Wang, Dong Yu, Wei Wei, Hao Zheng, Ming-Hua Liu, Long Ma, Li-Na Qin, Neng-Zhuang Wang, Jia-Xi Li, Jin-Jiang Wang, Xin-Ling Bi, Hong-Li Yan

**Affiliations:** 1 Center for Reproductive Medicine, Changhai Hospital, Naval Medical University, Shanghai, China; 2 Institute of Translational Medicine, Naval Medical University, Shanghai, China; 3 Clinical Research Center, Changhai Hospital, Naval Medical University, Shanghai, China; 4 Department of Dermatology, Changhai Hospital, Naval Medical University, Shanghai, China; 5 Department of Oncology, Tongren Hospital, Shanghai Jiao Tong University School of Medicine, Shanghai, China

**Keywords:** Junctional epidermolysis bullosa intermediate, *COL17A1*, Paternal uniparental disomy, Whole exome sequencing, Genetic counselling, Case report

## Abstract

**Background:**

Uniparental disomy (UPD) is a condition in which both chromosomes are inherited from the same parent, except for imprinting disorders. Uniparental isodisomy (UPiD) may result in a homozygous variant contributing to an autosomal recessive disorder in the offspring of a heterozygous carrier. Junctional epidermolysis bullosa intermediate (JEB intermediate) is an autosomal recessive inherited disease that is associated with a series of gene variants, including those of *COL17A1*.

**Case presentation:**

We report the first case of complete paternal UPiD of chromosome 10 harbouring a novel homozygous variant in *COL17A1*: c.1880(exon23)delG (p.G627Afs*56). This variant led to the clinical phenotype of junctional epidermolysis bullosa intermediate in a 5-year-old child. Trio-whole exome sequencing (Trio-WES) and in silico data analysis were used for variant identification, Sanger sequencing was performed for variant validation, and pathological examination was performed as the gold standard for phenotype confirmation.

**Conclusions:**

We recommend the use of WES as a first-tier test for the diagnosis of epidermolysis bullosa, especially for paediatric patients. Moreover, UPD events should be detected and analysed routinely through WES data in the future.

**Supplementary Information:**

The online version contains supplementary material available at 10.1186/s12920-022-01285-x.

## Background

Junctional epidermolysis bullosa intermediate (JEB intermediate, OMIM 226650), a subtype of JEB, is an autosomal recessive inherited disease with the clinical manifestation of blisters, mucosal involvement, atrophic scars, alopecia, nail dystrophy and dental abnormalities [1]. Several gene variants may contribute to JEB intermediate. One such variant is the gene collagen type XVII alpha 1 chain (*COL17A1*, NM_000494.4) variant that causes collagen XVII function defects and results in JEB intermediate.

Since JEB intermediate follows an autosomal recessive inheritance mode, it often occurs in a situation in which the proband’s parents are both heterozygous carriers. However, the situation of uniparental isodisomy (UPiD), a type of uniparental disomy (UPD) in which the two inherited identical homologues are from one parental chromosome, may result in a homozygous variant contributing to an autosomal recessive disorder in the offspring of a heterozygous carrier.

In recent years, next-generation sequencing (NGS) technology has become the prevailing method in the diagnosis of rare disorders. Particularly, the approach of whole-exome sequencing (WES) has been commercialized and widely used because of its less complex and more cost-effective nature. WES with bioinformatics support can identify variants in cases of epidermolysis bullosa (EB) [2] and detect UPD events [3] simultaneously through different algorithms. These abilities can improve the diagnostic efficiency and informatics for genetic counselling.

Here, we describe the first case of a complete paternal UPiD of chromosome 10 harbouring a novel homozygous mutation in *COL17A1* that leads to the clinical phenotype of the JEB intermediate in a 5-year-old child that was detected by WES and in silico data analysis.

## Case presentation

**Fig. 1:**
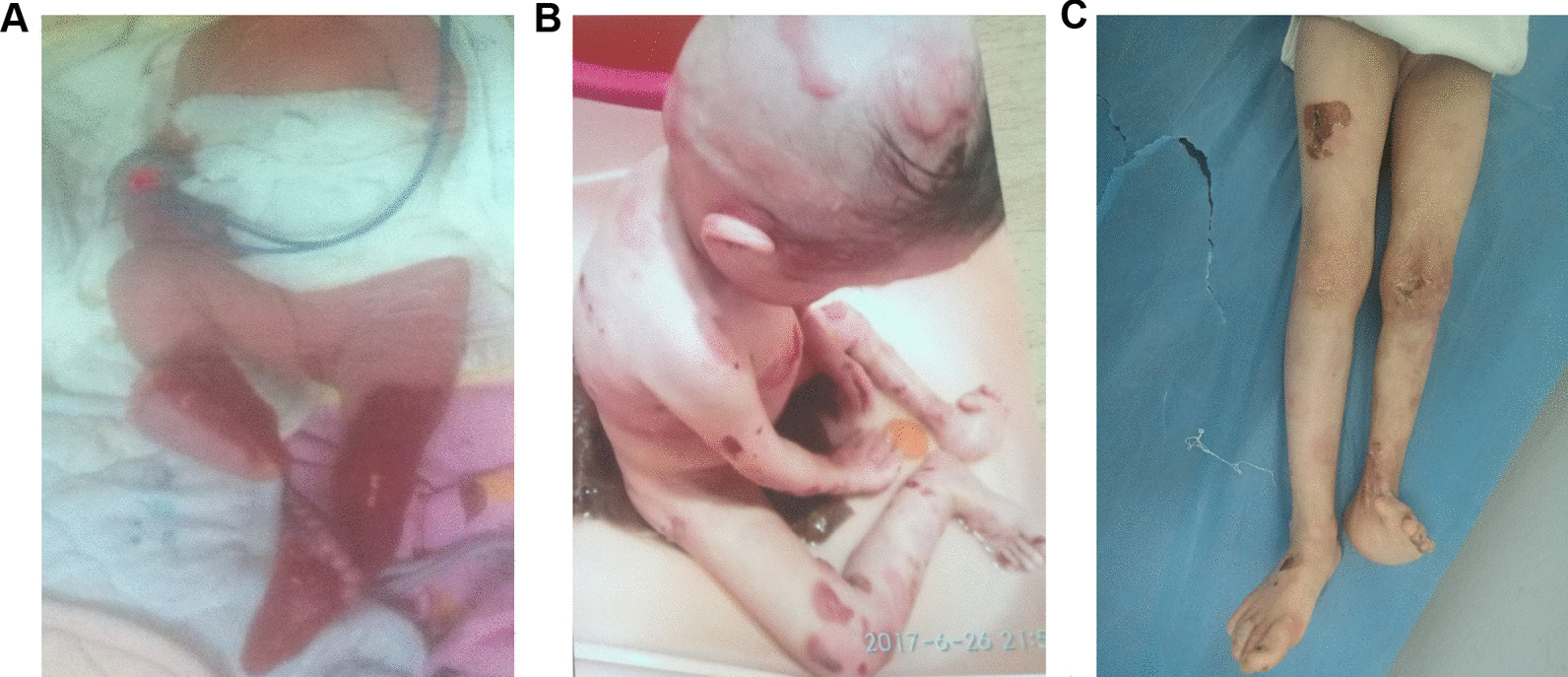
Clinical features, histopathological analyses and ultrastructural features of the proband. **A**. The neonatal features of the proband. **B**. The skin features of the proband at 2 years of age. **C**. Limb deformity and skin features of the proband at 5 years of age

The proband was born at term via spontaneous vaginal delivery with no shortness of breath or twitch. The Apgar scores were 10 at both 1 min and 5 min. A physical examination of the patient showed the following: a birth weight of 2,100 g, clear consciousness, poor reaction, smooth oral mucosa, extensive skin erosions of the extremities, especially on both of the lower legs, with excoriation and exposure of the blood vessels and fresh granulation tissue. The affected skin had a scalded skin appearance, especially the distal parts, and the affected area was approximately 10%. The left lower ankle joint was deformed, the left sole was folded parallel to the left lower extremity and thinner than the right lower extremity, the muscle tension of the limbs was slightly weak, the holding reflex was weakened, and the sucking reflex was normal. When the proband was 5 years old, she had the clinical characteristics of normal intelligence, systematic recurrent blister erosions, thick nails and stunted growth (Fig. 1 and Additional file 1: Fig. S1).

No unusual results were detected through laboratory examinations, including routine tests of blood, urine, hepatorenal and thyroid function. The patient’s parents presented no manifestations in their skin and other organs, and there was no history of consanguinity. Consent was obtained from the patient’s parents, and the research was performed according to the guidelines of the Helsinki Declaration.

**Fig. 2:**
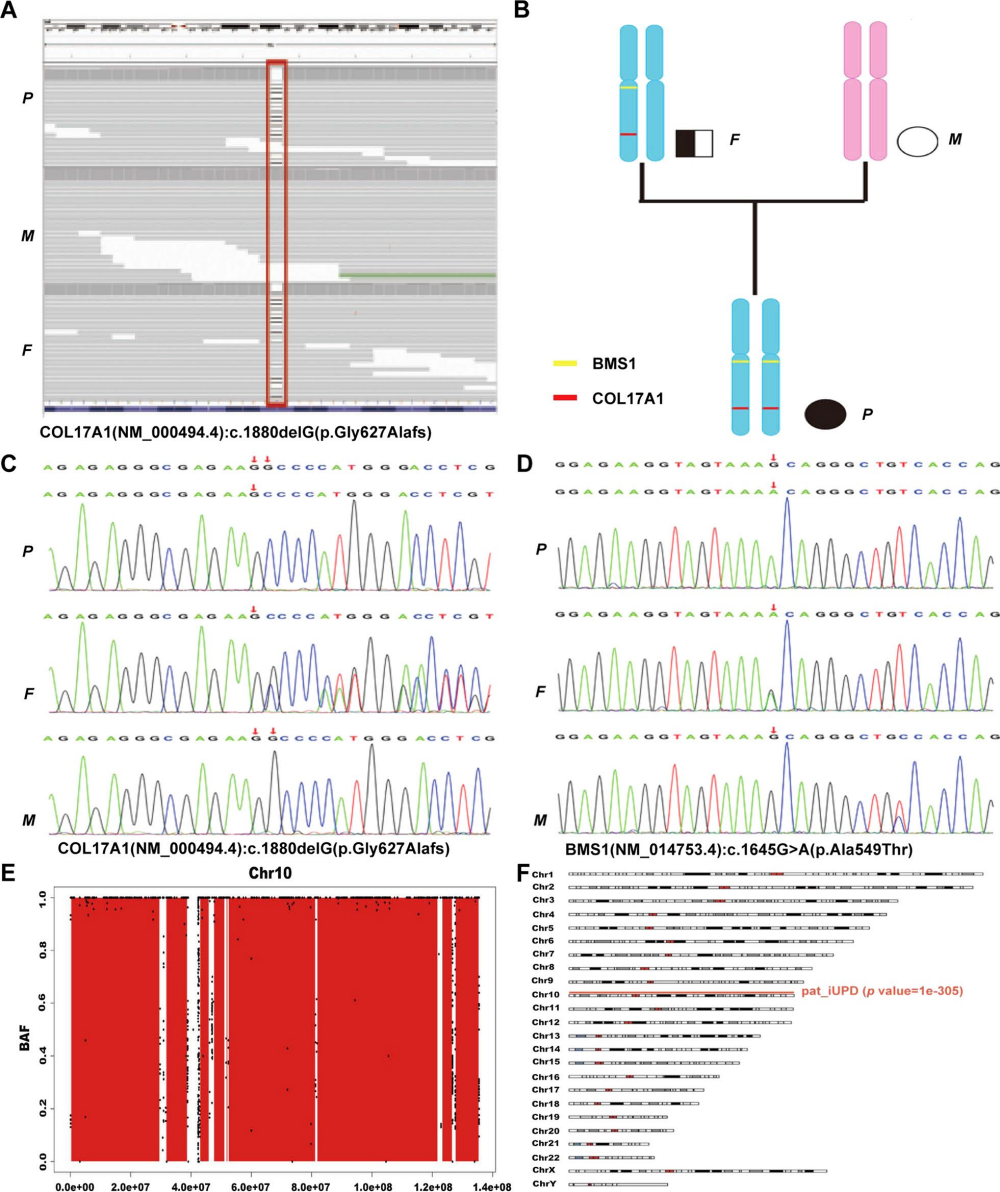
Trio-whole exome sequencing data analysis, validation and bioinformatics analysis. **A**. The filtering of the variant of COL17A1 according to ACMG guidelines, and the proband’s clinical features and the variant carriage status of the family members. **B**. The pedigree of the proband’s family. **C** and **D**. The verification of the mutations of COL17A1, c.1880(exon23)delG and BMS1: c.1645(exon10)G > A, through Sanger sequencing. The carriage status of the proband and her father and mother is homozygous, heterozygous and wild-type, respectively. **E**. The homozygosity (ROH) region in chromosome 10. **F**. The detection of UPD events through trio analysis using UPDio

Trio-WES was performed on the proband and her parents to identify the causative variants. The data analyses were performed (reference genome version hg19), and the filter VCFs were used as input by UPDio to detect UPD events based on MIE [3]. A total of 2215 SNVs/INDELs for this trio were identified, which were then annotated for the clinical interpretation according to the ACMG guidelines using the software Intervar [4] with reference to the web resources listed in the data availability section and 7 variants were identified as pathogenic/likely pathogenic (Additional file 2: Table S1). Considering that the proband’s parents had no clinical manifestations and that the inheritance patterns of the diseases correlated with the gene variants, the novel homozygous variant in *COL17A1* (NM_000494.4), c.1880(exon23)delG (p.G627Afs*56), was defined as likely pathogenic (PVS1 + PM2). The related disease was JEB intermediate (OMIM: 226650). This variant of *COL17A1* and another variant on chromosome 10 were further confirmed by Sanger sequencing (Fig. 2). Furthermore, the parent-offspring relationship was confirmed using the function relatedness of vcftools.

The patient’s WES data showed a run of homozygosity (ROH) region in chromosome 10, including the *COL17A1* gene, based on H3M2 (Fig. 2). ROH regions can be copy number neutral or show copy number loss. WES-based copy number analysis using ExomeDepth yielded normal results in the genomic region of *COL17A1*, thereby ruling out genomic copy number abnormalities. Trio analysis using UPDio showed a UPD event (paternal UPiD on chromosome 10) that included the *COL17A1* gene (Fig. 2). Collectively, these results indicated that the homozygous state of the patient’s c.1880delG *COL17A1* variant was due to paternal UPiD. That is, the proband’s two identical homologues of chromosome 10 were inherited from the same chromosome of the father. UPiD unmasked the heterozygous variant to cause the disease, which is an autosomal recessive inheritance mode.

**Fig. 3:**
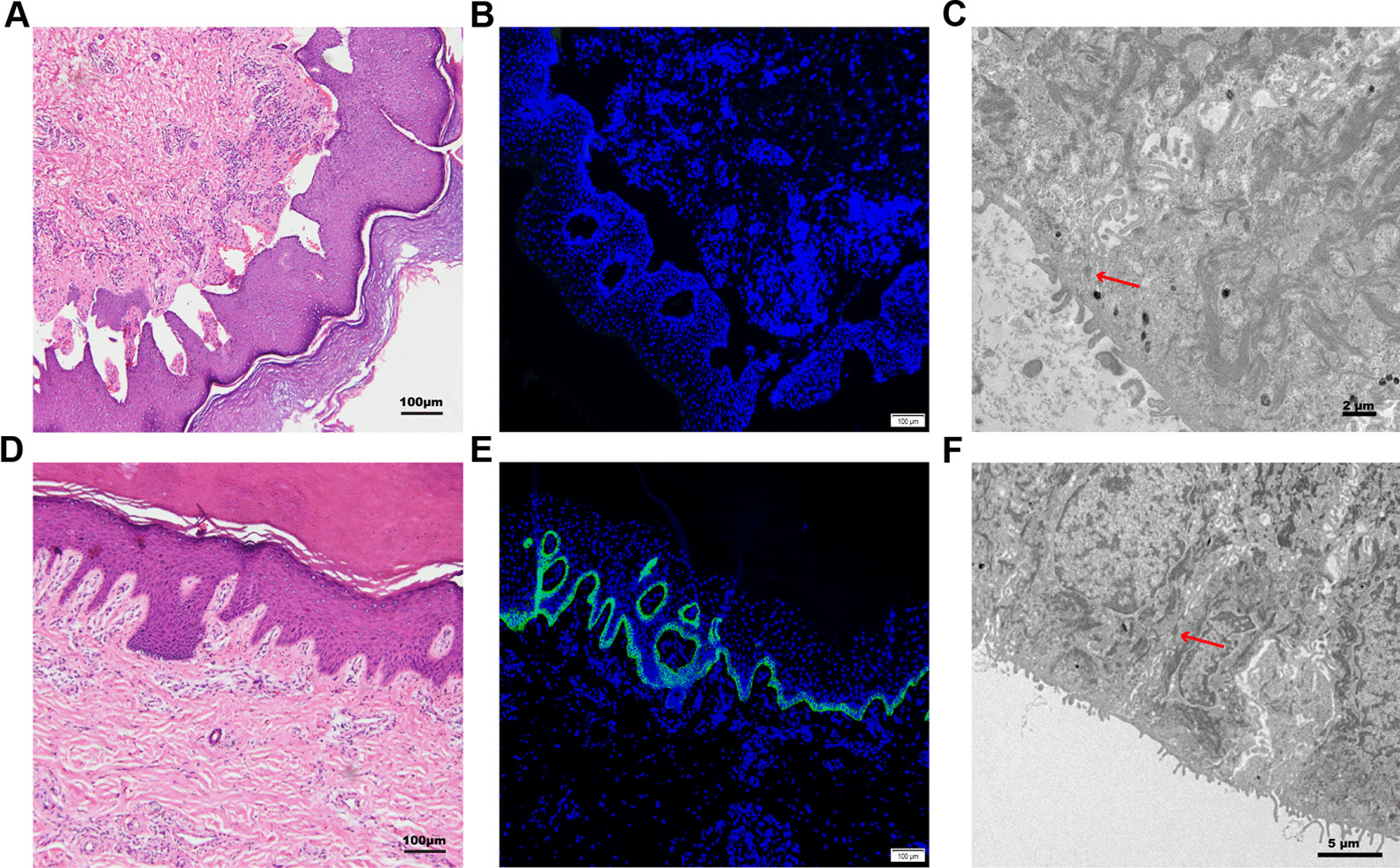
Histopathological analyses and ultrastructural features of the proband. **A** and **D**. Haematoxylin–Eosin staining of the skin tissue of the proband **A** and normal control tissue **D** of the proband. The connection between the dermis and epidermis was loosened, the interdermal cracks were enlarged and blisters had formed. **B** and **E**. immunofluorescence images of the proband **B** and a normal control **E**. Collagen XVII was linearly distributed between the dermis and epidermis of the normal control skin **E**, but the proband’s skin displayed Collagen XVII deficiency in the basal keratinocytes (**B**). The immunofluorescence figures were merged by the images captured at a magnification of 100× using ultraviolet filter and FITC filter. **C** and **F**. TEM showed the hemidesmosomes (arrows) were of lower density, had atypical structures and were decreased in number

The histopathology and ultrastructure results of the punch biopsy were analysed. The Haematoxylin–Eosin staining (HE staining) and immunofluorescence were observed using the Olympus-BX53 microscope (Olympus., Japan) with the software of Olympus cellSens Standard 1.17 (Olympus Ltd., Japan). The HE staining was imaged with the light filter by magnification of 100×, and immunofluorescence figures were merged by the images captured at a magnification of 100× using ultraviolet filter and FITC filter. The images of HE staining showed that the connection between the dermis and epidermis was loosened, the interdermal cracks were enlarged and blisters were formed (Fig. 3). Additionally, collagen XVII deficiency of the proband was demonstrated by immunofluorescence using *COL17A1* monoclonal antibody (Fig. 3). A Hitachi-7650 transmission electron microscope (Hitachi Ltd., Japan) was used for transmission electron microscopy (TEM) observation with an accelerating voltage of 80 kV, magnification of 100,000 times to 200,000 times. Through TEM observation, the hemidesmosomes were found to have lower density and atypical structures and were decreased in number (Fig. 3). All the parameters for the image acquisition in HE staining, immunofluorescence and TEM are the same avoiding threshold manipulation, expansion or contraction of signal ranges and the altering of high signals.

Because there is no specific symptomatic treatment or drugs, routine skin care had been performed on the patient since birth.

## Discussion and conclusions

In this case, trio-WES and bioinformatics analysis were used for causative gene filtration and aetiological diagnosis, and the results were validated by traditional gold standard immunofluorescence antigen mapping (IFM) analysis and ultrastructure observation.

JEB intermediate is inherited in autosomal recessive pattern; that is to say that a biallelic mutation may lead to pathogenic phenotype. In this study, trio-WES showed that the proband’s father was a heterozygous carrier of the *COL17A1* variant, and the gene sequence of the proband’s mother was wild-type. Furthermore, the Sanger sequencing results of the other variant of BMS1 ribosome biogenesis factor (*BMS1*), c.1645(exon10)G > A, in chromosome 10 were consistent with the previous results. These results imply that the inheritance of the chromosome 10 region of the proband follows a paternal UPD pattern. Then, H3M2 was used for ROH detection, and UPDio of the trio analysis confirmed the complete paternal UPiD of chromosome 10. The paternal UPiD of chromosome 10 of the proband unmasked the novel mutation in exon 23 of *COL17A1*, c.1880(exon23)del. This mutation results in a frame shift at the protein level that leads to the formation of a premature termination codon in *COL17A1*. Herein, to the best of our knowledge, this is the first case of JEB intermediate caused by complete paternal UPiD of chromosome 10 harbouring a novel mutation in exon 23 of *COL17A1*, c.1880(exon23)del.

In this case, the presumed pathogenic mechanism of action is recessive gene activation, and this has been reported as a causative factor for EB by other researchers. For example, the complete maternal isodisomy of chromosome 3 unmasked the heterozygous variation of the collagen type VII alpha 1 chain (*COL7A1*) that caused recessive dystrophic EB (RDEB) in a child without other phenotypic abnormalities [5, 6]. Additionally, the complete paternal isodisomy of chromosome 17 harbouring a homozygous variation of β4 integrin subunit (*ITGB4*) caused JEB with pyloric atresia (JEB-PA) [7]. Moreover, two cases of complete paternal UPiD of chromosome 1 resulted in JEB severe with a pathogenic variation in the laminin subunit beta 3 (*LAMB3*) [8] and laminin subunit gamma 2 (*LAMC2*) [9], respectively.

Previous studies have shown that chromosome UPD can be pathogenic. For example, UPD of chromosomes 6, 7, 11, 14, 15 and 20 may lead to imprinting disorders (e.g., transient neonatal diabetes, Silver-Russell syndrome, Beckwith-Wiedemann syndrome, Temple syndrome, Kagami syndrome, Prader-Willi syndrome, Angelman syndrome and pseudohypoparathyroidism type Ib) [10, 11]. However, there is no clinical evidence showing that UPD of chromosome 10 may cause imprinting disorder. To date, there have been only two reported cases of complete maternal UPiD of chromosome 10. One was derived from mosaic trisomy 10 and resulted in a male child born with no clinical phenotypes [11]. The other case was a 20‑month‑old female infant with severe and complex medical problems that could not be fully explained by gene mutations and recessive genes activated by UPiD [12].

According to the new edition of the clinical practice guidelines for the laboratory diagnosis of EB, paediatric and adult patients with skin fragility can be referred directly to a genetic diagnosis with NGS or Sanger sequencing [13]. The approach of WES has identified novel genes and multigene mutations in patients with EB even under the condition of IFM. Additionally, TEM could not identify the “candidate” gene, or such information is not available [2, 14–17]. Moreover, when WES is used for the diagnosis of EB, it reduces the need for diagnostic skin biopsies in EB and reduces laboratory costs. It also can detect mosaicism and allows for prenatal diagnosis, genetic counselling and predictive diagnosis for partners with a carrier status [13]. Additionally, as blood samples are easily acquired, relatively non-invasive and easy to ship, the test on blood samples makes centralized EB examination and analysis possible. Thus, it may markedly reduce the cost of testing and staff required, which would make the test available for more patients.

It is worth mentioning that although UPD can be analysed through WES data, third-party laboratories rarely analyse UPD events. Additionally, there are few reports on them [3]. The incidence of UPD has been estimated to be approximately 0.029% in newborns [18]. According to the literature, only one UPiD was found in 676 healthy subjects [19]. For this case, with the diagnosis of UPiD as the cause of JEB intermediate, the recurrence risk of the family reduces from 25% to less than 1%, which may change the genetic counselling for future pregnancy and prenatal diagnosis.

The parents of the patient plan for another pregnancy and will keep in touch with us for genetic counselling. We are trying to develop new methods for the noninvasive prenatal diagnosis on the basis of maternal plasma and in silico analysis, including UPiD analysis.

In conclusion, this is the first case of complete paternal UPiD of chromosome 10 harbouring a novel homozygous mutation in *COL17A1* that leads to the clinical phenotype of JEB intermediate. However, there are no specific therapeutic methods to improve the patient’s quality of life. The only option is to make the diagnosis as early as possible, avoid overtreatment and relieve the patient’s pain. We recommend the approach of WES to be used as a first-tier test for the diagnosis of EB, especially for paediatric patients. Moreover, UPD events should be detected and analysed routinely through WES data. In the future, more algorithms should be explored to improve the effectiveness and diagnostic value of WES.

## Supplementary Information

**Additional file 1**. Clinical features of the skin and limb deformity of the proband.

**Additional file 2**. Table of identified variants annotated as pathogenic/likely pathogenic for the trio.

## Data Availability

The variant NM_000494.4(*COL17A1*):c.1880del (p.Gly627fs) has been deposited in the ClinVar (https://www.ncbi.nlm.nih.gov/clinvar/) with the accession number of SCV002506514. The whole exome sequencing data generated during the current study and related phenotype information have been deposited in the Genome Sequence Archive (GSA). The direct web links to this dataset is https://ngdc.cncb.ac.cn/search/?dbId=hra&q=HRA001878. For the access of the raw data, the authors Hong-Li Yan (hongliyan@smmu.edu.cn) and Yao Wang (wangyaowxfy@163.com) can be contacted. The web resources for reference are as follows: 1000 Genomes Project, http://www.1000genomes.org/; ANNOVAR, http://annovar.openbioinformatics.org/; ClinVar, https://www.ncbi.nlm.nih.gov/clinvar/; CLINVITAE, http://clinvitae.invitae.com/; dbNSFP, https://sites.google.com/site/jpopgen/dbNSFP; dbscSNV, https://sites.google.com/site/jpopgen/dbNSFP; dbSNP, http://www.ncbi.nlm.nih.gov/SNP; Ensembl, http://www.ensembl.org/; Exome Aggregation Consortium (ExAC) Browser, http://exac.broadinstitute.org; GERP +  + , http://mendel.stanford.edu/SidowLab/downloads/gerp/; GWASdb, http://jjwanglab.org/gwasdb; HGMD, http://www.hgmd.org; InterVar, https://github.com/WGLab/InterVar; MedGen, https://www.ncbi.nlm.nih.gov/medgen/; NHLBI Exome Sequencing Project (ESP) Exome Variant Server, http://evs.gs.washington.edu/EVS/; OMIM, http://omim.org/; OrphaNet, http://www.orpha.net/; PolyPhen-2, http://genetics.bwh.harvard.edu/pph2; RefSeq, http://www.ncbi.nlm.nih.gov/refseq; RepeatMasker, http://www.repeatmasker.org/; SIFT, http://sift.jcvi.org/; UCSC Genome Browser, http://genome.ucsc.edu; wIntervar, http://wintervar.wglab.org/.

## Abbreviations

JEB intermediate: Junctional epidermolysis bullosa intermediate
EB: Epidermolysis bullosa
RDEB: Recessive dystrophic epidermolysis bullosa
JEB-PA: Junctional epidermolysis bullosa with pyloric atresia
JEB severe: Junctional epidermolysis bullosa severe
*LAMC2*: Laminin subunit gamma 2
*COL17A1*: Collagen type XVII alpha 1 chain
*COL7A1*: Collagen type VII alpha 1 chain
*ITGB4*: β4 Integrin subunit
*LAMB3*: Laminin subunit beta 3
*BMS1*: BMS1 ribosome biogenesis factor
UPD: Uniparental disomy
UPiD: Uniparental isodisomy
NGS: Next-generation sequencing
WES: Whole exome sequencing
ROH: Run of homozygosity
HE staining: Hematoxylin–eosin staining
TEM: Transmission electron microscope
IFM: Immunofluorescence antigen mapping
