# Supplementary material for: First case report of complete paternal isodisomy of chromosome 10 harbouring a novel variant in COL17A1 that causes junctional epidermolysis bullosa intermediate
Source: BMC Med Genomics. 2022 Jun 18;15:136. doi: 10.1186/s12920-022-01285-x (PMC9206295; doi:10.1186/s12920-022-01285-x)
Supplement: Supplementary file 1 — Additional file 1. Clinical features of the skin and limb deformity of the proband. [file 12920_2022_1285_MOESM1_ESM.docx]

**Additional file 1: Fig. S1**

**
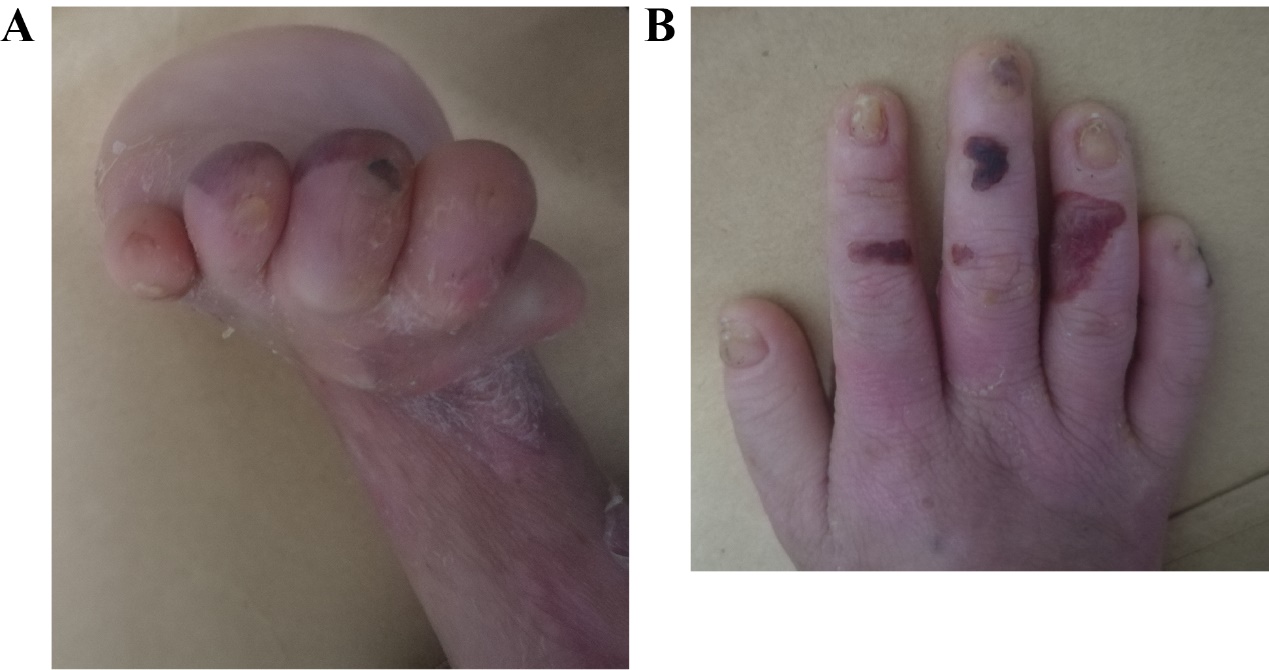
**

Additional file 1: Fig. S1 Skin features and limb deformity of the proband. A. Left lower limb deformity with blisters and erosions of the skin and thick nails. B. Right hand with blisters and thick nails.
